# Working With Communities to Support Minority Caregivers: The PRISMA Health REACH Expansion Project

**DOI:** 10.1093/geroni/igaa057.3099

**Published:** 2020-12-16

**Authors:** Mindi Spencer, Maggi Miller, Diana Jahries, James Davis

**Affiliations:** 1 University Of South Carolina, Columbia, South Carolina, United States; 2 Center for Success in Aging, PRISMA Health, Greenville, South Carolina, United States

## Abstract

In 2019, the NIH presented the results of its two-year visioning process to advance the science of minority health and health disparities. The PRISMA Health REACH Expansion (PH-REACH E) is an innovative, community-academic partnership between a hospital memory clinic, meal delivery service, research university, and low-income health clinic. The purpose is to: 1) increase the dementia capability of community-based programs, 2) offer caregivers of persons with dementia the REACH intervention, and 3) identify and connect racial minority and/or rural residents with services to promote health and well-being in older adulthood. This presentation will detail the PH-REACH E framework and present program results, which include improved caregiver outcomes (e.g., reduced burden, increased self-efficacy, reduced depression) and enhanced dementia capability (e.g., increased dementia knowledge) of partner organizations. This program illustrates some key recommendations of the NIH – community engagement in intervention adaptation, multisectoral collaboration, and promoting systems-level change to reduce health disparities.

